# The Role of Cell Adhesion Molecule 1 (CADM1) in Cutaneous Malignancies

**DOI:** 10.3390/ijms21249732

**Published:** 2020-12-20

**Authors:** Yu Sawada, Emi Mashima, Natsuko Saito-Sasaki, Motonobu Nakamura

**Affiliations:** Department of Dermatology, University of Occupational and Environmental Health, 1-1 Iseigaoka, Yahatanishi-ku, Kitakyushu 807-8555, Japan; e-mashima@med.uoeh-u.ac.jp (E.M.); natsuko-saito@med.uoeh-u.ac.jp (N.S.-S.); motonaka@med.uoeh-u.ac.jp (M.N.)

**Keywords:** CADM1, skin, squamous cell carcinoma, melanoma, lymphoma, Merkel cell carcinoma

## Abstract

Cell adhesion ability is one of the components to establish cell organization and shows a great contribution to human body construction consisting of various types of cells mixture to orchestrate tissue specific function. The cell adhesion molecule 1 (CADM1) is a molecule of cell adhesion with multiple functions and has been identified as a tumor suppressor gene. CADM1 has multifunctions on the pathogenesis of malignancies, and other normal cells such as immune cells. However, little is known about the function of CADM1 on cutaneous cells and cutaneous malignancies. CADM1 plays an important role in connecting cells with each other, contacting cells to deliver their signal, and acting as a scaffolding molecule for other immune cells to develop their immune responses. A limited number of studies reveal the contribution of CADM1 on the development of cutaneous malignancies. Solid cutaneous malignancies, such as cutaneous squamous cell carcinoma and malignant melanoma, reduce their CADM1 expression to promote the invasion and metastasis of the tumor. On the contrary to these cutaneous solid tumors except for Merkel cell carcinoma, cutaneous lymphomas, such as adult-T cell leukemia/lymphoma, mycosis fungoides, and Sézary syndrome, increase their CADM1 expression for the development of tumor environment. Based on the role of CADM1 in the etiology of tumor development, the theory of CADM1 contribution will desirably be applied to skin tumors according to other organ malignancies, however, the characteristics of skin as a multicomponent peripheral organ should be kept in mind to conclude their prognoses.

## 1. Introduction

The classical phenomenon of differential adhesion is hypothesized to develop a better understanding of how cells connect with each other, establish tissue rearrangement, and organize the human body [1]. As a part of this contribution, cell adhesion is one of the established natures for constructing the human body.

Cell adhesion involves various cell’s dynamic function, such as movement and interaction with other cells. These cell adhesion components have developed stylishly to accelerate the potential of cell function. One of the adhesion molecules, the cell adhesion molecule 1 (CADM1) has multiple functions as a member of the immunoglobulin superfamily of transmembrane glycoproteins. This adhesion molecule has been identified as a suppressor gene of tumors, and CADM1-transfected large lung cell cancer cells showed decreased development of the tumor [2]. After this novel finding, there are many reports focused on CADM1 function in malignancies, and other cells such as immune cells have been postulated [2,3,4,5]. Various normal tissues and organs express CADM1 under physiological condition, such as mast cells, neurons, and vascular endothelial cells [6,7,8]. On the contrary to these normal cells, various malignancies take advantage by dysregulation of this cell adhesion component for the development of their tumor microenvironment. 

Skin is the most outer layer organ, and consists of various cell components with tight junctions and cell adhesion to communicate with other cells to protect against the external environment. These characteristics are a first line of defense by establishing the tightly organized skin surface against the external environment, therefore the skin has a robust structure to protect the human body from the external environment. For example, skin surface disruption by toxic epidermal necrolysis causes life-threatening clinical outcomes [9]. These clinical observations reveal how skin plays an important role to defend against the external environment. Therefore, skin seems to be one of the organs most contributed to by these cell adhesion molecules, however, little is known about the cutaneous cells and skin malignancies. In this review, we focused on the contribution of CADM1 on the skin and the development of cutaneous malignancies. There is a limited amount of research focused on CADM1 in dermatology fields, therefore we also mention some contemporary advances of CADM1 research to be expected for future investigation in dermatology. Finally, the clinical application of CADM1-targeted therapy and the usefulness of CADM1 as a biomarker to evaluate disease severity and progression are also described.

## 2. The Role of CADM1 in Cell Types

CADM1 encodes an immunoglobulin-like cell adhesion molecule consisting of three loops of immunoglobulin [2]. The CADM1 ectodomain acts as an intercellular adhesion mediated by homophilic or heterophilic trans-interaction in each cell [10]. On the other hand, the CADM1 cytoplasmic domain consists two conserved protein-interaction modules, the submembranous protein 4.1-binding motif (protein 4.1-BM), and the type II PDZ-binding motif (PDZ-BM) [2]. Protein 4.1-BM combines CADM1 to the intracellular actin cytoskeleton structures [11]. PDZ-BM is a membrane-associated guanylate kinase homolog (MAGUK) that interacts through PDZ (PSD-95, Discs large and ZO-1) domains [12], and induces various cellular functions. For instance, TIAM1 (T-lymphoma invasion and metastasis 1) bears a type II PDZ domain and CADM1 promotes Tiam1-mediated Rac activation, which are involved in cell migration [13]. The detailed functions of intracellular domain of CADM1 are little known, therefore it is desirable for it to be clarified in various cells and tumor cells.

**Figure 1:**
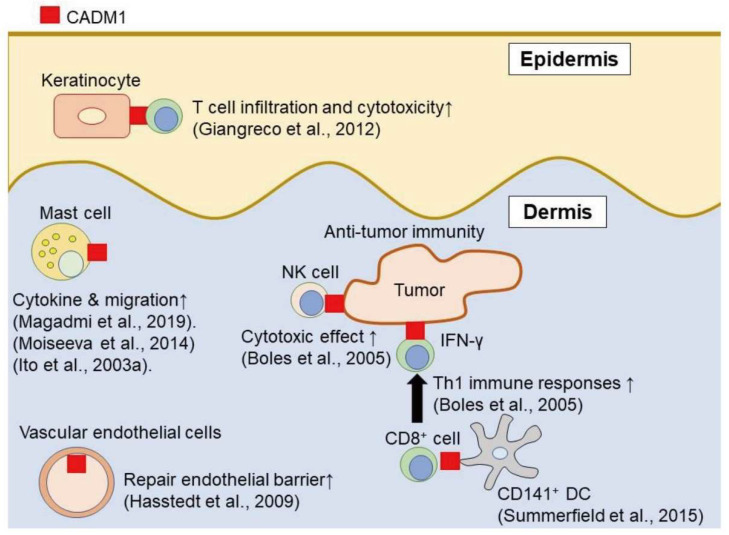
The involvement of cell adhesion molecule 1 (CADM1) in cutaneous cell function.

CADM1 contributes to the interaction in each individual cell and other types of cells. In addition, CADM1 is known to act as a scaffolding molecule for various cells to support cell motility. In this section, we mainly focused on the CADM1 function in skin components or related cells, such as keratinocyte, vascular endothelial cells, mast cells, dendritic cells, natural killer (NK) cells, CD8^+^ cells, and neuron cells (Figure 1).

This scheme represents CADM1 involving cell functions in the skin. CADM1 on keratinocytes acts as a scaffolding molecule for immune cells. CADM1 on vascular endothelial cells promotes the repair of the endothelial barrier. CADM1 on immune cells, such as dendritic cells (DC), T cells, NK cells and mast cells, contributes to the development of their immune functions.

### 2.1. Keratinocytes 

Keratinocytes are the major components in the epidermis and exert a defense function against the external environment. Keratinocytes express CADM1, which contributes to CD8^+^ cell infiltration into the epidermis in patients with alopecia areata. Overexpressed-CADM1 in human epidermal keratinocytes increases adhesion of cytotoxic T cells and enhances their cytotoxic function. Epidermal overexpressed-CADM1 transgenic mice had an enhanced autoimmune alopecia reaction, suggesting that CADM1 supports adhesion ability of lymphocytes [14] and acts as a scaffolding molecule for other cells to promote inflammation in the epidermis.

### 2.2. Mast Cells

A mast cell contains granules with enriched histamine and heparins and is located in the connective tissues [15]. A study has revealed the diverse and multiple functions of mast cells in skin diseases [16,17]. CADM1 is expressed in human mast cells [18], and the secretion of inflammatory cytokines is enhanced by the adhesion to sensory neurons in a CADM1-dependent manner [19]. CADM1 also controls mast cell migration and extra-matrix adhesion, and contacts other cells in an actin cytoskeleton assembling-dependent manner [6,20].

### 2.3. Dendritic Cells

Dendritic cells are representative antigen presentation cells and masters of orchestrating of immune responses. Furthermore, dendritic cells determine the direction of immune responses after the exposure to external antigens [13,21,22]. CADM1 is expressed on cutaneous CD141^+^ dendritic cells [23], which exhibit a high expression of toll-like receptor 3, and production of inflammatory cytokines, IL-12p70, and IFN-β. Furthermore, this DC subset has a greater ability for the induction of Th1 immune responses [24]. 

### 2.4. NK Cells and CD8^+^ Cells

NK cells and CD8^+^ T cells are lymphocytes in the circulating peripheral blood that recognize tumor cells. NK cells respond to inflammatory chemokines and other stimulations released by damaged tissues [25]. The activated NK cells show cytotoxic activity and produce inflammatory cytokines and chemokines to amplify immune responses [26]. During antigen presentation by antigen-presenting cells in acquired immunity, the activated cytotoxic CD8^+^ T cells in the inflammatory site accelerate antigen-specific antitumor immunity [27]. CADM1 has the potential to activate tumor lysates by NK cells and strong IFN-γ production by CD8^+^ T cells [28], indicating that CADM1 acts as the communication molecule for immune cells to invoke their immune ability against tumors.

### 2.5. Neuron Cells

Neurons involve various skin physiological and pathological functions, leading to the development of various skin diseases [29]. Nerves also exert various cells to promote physiological and pathological functions [30]. CADM1 develops synaptic adhesion which drives synapse assembly [7].

## 3. CADM1 and Human Cutaneous Malignancies

CADM1 was first noticed as a suppressor gene of tumor in a non-small cell lung cancer, which was found as a favorable clinical factor in malignancies of solid tumors [2]. In general, as the first step of invasion and metastasis of the solid tumors, the fragility of adhesion ability in the tumor is necessary for release from the primary sites. Therefore, it is assumed that the invasion and metastasis is closely related with the loss of CADM1 expression. Actually, CADM1 expression in lung cancer tissues is inversely correlated with clinical stage progression [3,4]. On the contrary to the solid malignancies, CADM1 exhibits the opposite clinical behavior in bone marrow-derived tumors. Adult T cell leukemia/lymphoma (ATLL) tumor cells highly upregulate CADM1 [5]. As one of the mechanisms, the expression of CADM1 on ATLL cells contributes to infiltration and the adhesion ability to vessels and the skin to form nodules and tumors. In this review, we focused on the role of CADM1 in the development of cutaneous malignancies and discussed the difference between cutaneous solid tumors and lymphomas (Table 1).

### 3.1. Cutaneous Squamous Cell Carcinoma

Cutaneous squamous cell carcinoma is a malignancy derived from keratinocytes in the epidermis, and the importance of increasing incidence rate is highlighted for clinicians [42,43]. The metastatic form of cutaneous squamous cell carcinoma exhibits an unfavorable prognosis due to there being no effective treatment against advanced squamous cell carcinoma [44,45].

Decreased CADM1 expression in cutaneous squamous cell carcinoma demonstrates poor survival rates [35]. In addition, a genome-wide study also suggested that CADM1 plays a role in tumor development [36]. Therefore, these results indicate that CADM1 is a key molecule in cutaneous squamous cell carcinoma to develop the advanced form by inhibition of CADM1 expression of the tumor. However, the tumor differentiation is also one of the prognostic factors in squamous cell carcinoma [44], and is closely related with the degree of cell adhesion molecules [46]. The actual role of CADM1 in squamous cell carcinoma is largely unknown, however, the degree of CADM1 expression in squamous cell carcinoma might also affect the infiltration of inflammatory cell migration into the tumor. Squamous cell carcinoma is a keratinocytes origin malignancy, and the reduction in CADM1 might impair the adhesion ability of lymphocytes [14], due to the loss of the scaffolding molecule for immune cells to promote inflammation in the epidermis. To clarify the detailed clinical impact of CADM1 on the development of cutaneous squamous cell carcinoma, large scale statistical analysis is needed to clarify whether CADM1 is an independent prognostic factor in cutaneous squamous cell carcinoma in the future analysis.

### 3.2. Malignant Melanoma

Melanoma is a melanocyte-derived malignancy with an unfavorable and severe life-threatening clinical behavior because of the characteristics of its malignancy in addition to the lack of radical treatment [47,48]. Current immune checkpoint inhibitors and v-raf murine sarcoma viral oncogene homolog B1 (BRAF)-targeted treatments develop their clinical outcomes. However, these do not reach satisfactory levels in their clinical outcomes. The average expression levels of CADM1 mRNA and protein in melanoma are significantly decreased compared with dysplastic nevi lesions and normal skin [49]. The tumor invasion and metastasis are a critical factor relating to an unfavorable clinical behavior in patients with malignant melanoma. It activates cell migration and the invasion of malignant melanoma by suppressing CADM1 expression [31]. There is a significant reduction in the expression of CADM1 in advanced melanoma compared to dysplastic compound melanocytic nevi [50]. The CADM1-transfected melanoma cells are significantly suppressed in their growth in vitro, and the ability of invasion is also reduced [32]. Increased expression of CADM1 results in a significant inhibition of motility and invasiveness of melanoma cells [33]. The survival of melanoma is significantly decreased in reduced expression of CADM1 patients with melanoma or harboring methylated CADM1, indicating that the epigenetic modification of CADM1 by hypermethylation is also an important factor in the pathogenesis of melanoma [34]. 

### 3.3. Cutaneous Lymphoma

#### 3.3.1. ATLL

ATLL is the human T cell lymphotropic virus type I (HTLV-1) associated with a malignancy of mature CD4^+^ T cells [51,52]. Based on the severity, organ involvement, and the number of abnormal lymphocytes, ATLL is divided into four clinical categories according to Shimoyama’s classification: acute, lymphoma, chronic, and smoldering types [52]. Skin involvement is observed in approximately 50% of ATLL patients; the evaluation of skin lesions is useful to estimate their prognosis [53,54,55]. While the acute and lymphoma types of ATLL exhibit unfavorable clinical behavior [56,57,58], the chronic and smoldering types are indolent and can usually be managed with “watchful waiting” until the acute crisis progression [59]. CADM1 is known to contribute to the development of ATLL. The frequency of CADM1^+^ T cells positively correlates with abnormal lymphocytes in the peripheral blood of ATLL patients. In addition, over 1% of CD4^+^CADM1^+^ cells show a significantly positive correlation with the copy number of HTLV-1 provirus in HTLV-1 carriers and ATLL patients [60]. Whole-genome microarrays in patients with acute ATLL identified that the expression of CADM1 increased more than 30-fold. The insertion of CADM1 promotes ATLL cells to cause aggregation and adhesion to vascular endothelial cells, suggesting that CADM1 is a biomarker for acute ATLL and its involvement in tumor invasion [5]. In experiments using immunodeficient mice, highly CADM1-expressed ATLL cells activated tumor formation and aggressive infiltration of various organs, suggesting that CADM1 plays an important role in the tumor growth and invasion into organs of ATLL cells [38]. Furthermore, CADM1 directly regulates Rac activity interactions, resulting in lamellipodia formation, which may lead to the tissue infiltration of leukemic cells in ATLL patients [10].

#### 3.3.2. Mycosis Fungoides

Mycosis fungoides are an epidermotropic T lymphocyte which infiltrate cutaneous T cell lymphoma [51,61,62]. Mycosis fungoides exhibit a basic indolent clinical behavior, with slow progression from patches to a more-infiltrated form of plaque, and eventually develops into nodules and tumors [51]. Therefore, the prognosis of patients with mycosis fungoides depends on the clinical stages, the skin lesions, and the extended systemic organ lesions [51]. Regarding the contribution of CADM1 on the pathogenesis of mycosis fungoides, our study has revealed that the survival rate in mycosis fungoides is significantly lower in patients with high CADM1-expressed groups. Therefore, CADM1 expression in mycosis fungoid tumor cells is negatively related to the prognosis of mycosis fungoides [39]. In addition, another study has revealed a more precise and detailed analysis of the contribution of CADM1 on mycosis fungoides [40]. They investigated CADM1 expression in mycosis fungoides tumor cells to identify its utility as a diagnostic marker for mycosis fungoides. CADM1 is expressed in mRNA in infiltrating lymphocytes into the dermis in patients with mycosis fungoides, although no CADM1 expression could be detected in those of patients with inflammatory skin diseases, suggesting it as a distinguished tool for an early stage of mycosis fungoides compared with inflammatory skin diseases [40]. 

#### 3.3.3. Sézary Syndrome

Sézary syndrome is defined as a leukemic form of cutaneous T cell lymphoma, showing clinical manifestations as erythroderma, pruritus, adenopathy, and circulating CD4^+^ atypical cells [61,62,63]. Only one study has reported the involvement of CADM1 in Sézary syndrome, however, their analyses suggest to us an important insight of CADM1 roles in cutaneous lymphoma. In their Sézary syndrome patient series analysis, CADM1 was expressed in patients with progressive type Sézary syndrome. Sézary syndrome (SS) patients with high frequency of peripheral CADM1^+^ cells show an unfavorable clinical behavior. The expression of CADM1 is not only an activation marker of SS tumor cells, but also the possible involvement of CADM1 as a scaffolding molecule in the epidermotropic infiltration of tumor cells into epidermal keratinocytes and penetration through the basement membrane zone.

## 4. Merkel Cell Carcinoma

Merkel cell carcinoma (MCC) is a rare cutaneous malignancy derived from a neuroendocrine origin of the mechanoreceptor unit of the skin with an aggressive clinical course [64]. Merkel cell carcinoma clinically presents as a flesh-colored or violaceous intracutaneous nodule with rapid growth [65,66]. Immune checkpoint inhibitor, anti-PD-L1 antibody, shows clinical efficacy for the treatment of advanced Merkel cell carcinoma [67], however, these treatments could not gain enough therapeutic effect. Cell adhesion molecule expressions relates with the development of outside spreading of this tumor [37]. The higher CADM1 expression in Merkel cell carcinoma is significantly correlated with the decreased overall survival, and one case has been identified where CADM1 is associated with hypermethylation of the promoter region [37]. Merkel cell polyomavirus is closed related to the pathogenesis of Merkel cell carcinoma, and Merkel cell polyomavirus-related MCCs show a significantly lower expression of CADM1, suggesting that high expression of CADM1 in Merkel cell carcinoma is significantly related to an unfavorable clinical outcome.

### 4.1. Advances of CADM1 Research in Non-Dermatological Fields

To determine the future direction of CADM1 research in dermatology, CADM1 research in the non-dermatological fields will be helpful to understand the future direction of CADM1 research. Contemporary studies have identified the role of CADM1 in various tumor progression, such as esophageal cancer [68], colon cancer [69], breast cancer [70], bladder cancer [71], and ovarian cancer [72]. In addition, research has also revealed the unexpected roles of CADM1. 

Several studies have identified the protective role of CADM1 in the body. CADM1 is involved in renal function and expresses in the renal distal tubules [73]. The reduced CADM1 is correlated with tubulointerstitial and tubular injuries and increased serum creatinine and BUN. Apoptosis of renal tubular epithelial cells is positively related to the CADM1 reduction. In addition, CADM1 silencing treatment enhances apoptosis of renal tubular epithelial cells in vitro. The decreased CADM1 expression causes apoptosis of renal tubular epithelial cells and promotes renal tubulointerstitial and tubular injuries, resulting in the development of chronic kidney disease. Another study showed the importance of CADM1 in cell survival [74]. The expression of CADM1 is observed in pulmonary epithelial cells, and CADM1 silencing treatment increases the apoptotic cells by TUNEL assays. Increased expression of CADM1 contributes to cell survival. 

Another study showed the possible beneficial impact of CADM1 in modulating intestinal barrier function [75]. Overexpression of CADM1 shows an increased Claudin-1 expression, while the silencing CADM1 improves the intestinal barrier function. Claudin-1 is responsible for barrier function in the skin [76,77], therefore CADM1 in keratinocytes is expected to exhibit a positive regulation in the skin barrier and have a beneficial impact on skin barrier-related diseases [78]. However, the actual impact of CADM1 in epidermal barrier function remains unclear.

CADM1 has been reported to relate to the metabolic system [79,80]. The expression of CADM1 was upregulated in an obesity mouse model. CADM1 in neurons impairs the gain of body weight while promoting energy consumption [79]. CADM1 reduction in the hippocampus and hypothalamus promoted weight loss. In addition, CADM1 deficiency in excitatory neurons increases leptin sensitivity and bone mass reduction, such as bone mineral content, femoral length, and bone strength [80]. 

Several parts of novel CADM1-mediated signal pathways have also been identified in the current research [81,82]. CADM1 is involved in the Hippo pathway core kinases, MST1/2 and LATS1/2, leading to the enhancement of YAP1 phosphorylation and downmodulated downstream pathway in lung adenocarcinoma cell line [81]. The estimated prognostic impact of CADM1 is currently roughly estimated, therefore the prognosis as to whether they are solid malignancies or hematological malignancies, different types of origin tumors, might explain the differences.

Another study has identified CADM1-mediated cell spreading mediated by PI3K [82]. PI3K inhibitors, Wortmannin and LY294002, inhibit cell spread in HEK293, although there are no inhibitory effects by JAK, MAPK, and NF-KB inhibitors. CADM1-mediated PI3K activation promotes Akt and Rac1 downstream, which enhance cell spreading. As the detailed mechanisms, CADM1 indirectly interacts with a PI3K subunit p85 by creating a protein complex with Dlg and MPP3 through the direct binding with PDZ-BM of CADM1 and is involved in cell spreading. Interestingly, this phenomenon is only observed under a high cell density, indicating that there are some mechanisms to activate this CADM1 function. In addition, this pathway is helpful to obtain a better understanding of CADM1-mediated cell motility by Rac activation in various cells, because Rac in almost all cells is mediated by PI3K activation [83,84,85].

### 4.2. CADM1 as a Biomarker

This section describes the usefulness of CADM1 as a biomarker to evaluate the disease progression and severity in several diseases, such as ATLL [86,87], cervical cancer [88], and chronic kidney disease [89]. 

Although soluble interleukin-2 receptor α (sIL-2Rα) is known to predict the progression of ATLL, plasma-soluble CADM1 was reported as a biomarker for aggressive ATLL [86]. Serum-soluble CADM1 becomes a biomarker for monitoring therapeutic efficacy to chemotherapy and the evaluation of ATLL relapse.

Another study has investigated flow cytometric analysis to identify CADM1 expression on CD4^+^ cells to predict the future risk of aggressive-type development [87]. Less than 25% CADM1 expression showed that no apparent progression of clinical disease was observed. However, cases of CADM1 expression over 25% and less than 50% showed that 55.5% of patients advanced from asymptomatic to smoldering type of ATLL. Furthermore, among CADM1 expression cases of over 50%, 28.4% of patients received systemic chemotherapy at three years. The percentage of the CD4^+^ CADM1^+^ cells is useful for estimating the progression of the disease.

The usefulness of plasma CADM1 methylation as a metastasis biomarker in cervical cancer has been reported [88]. Plasma levels of methylated CADM1 are increased in patients with advanced cervical cancer. 

CADM1 is also reported to be a possible biomarker for chronic kidney disease [89]. The expression of CADM1 is observed on renal tubular epithelia. A total of 44 patients (35%) had elevated serum CADM1 concentration in chronic kidney disease, suggesting that CADM1 is a marker for evaluating the damage in tubulointerstitial tissues in chronic kidney disease.

Among solid tumors, melanoma and squamous cell carcinoma have been known to be the diseases in which CADM1 can be utilized as a biomarker. The expression of CADM1 is reduced in melanoma above 1 mm in thickness [50]. In esophageal squamous cell carcinoma, CADM1 expression is related with the tumor development and the advanced tumor–node–metastasis stage [68]. Therefore, it is possible that CADM1 could be used as the biomarker for other cutaneous malignancies to estimate the disease progression, which should be explored in future.

### 4.3. Epigenetic Modification of CADM1

The mechanisms of phenotypic plasticity regulation and the cell capacity against changing its state in response to external stimuli had been largely unknown for a long time. The traditional central dogma of biological states is that DNA is thought to transcribe to RNA, subsequently leading to translation into proteins, cellular processes, and functions [90]. However, the diverse responses to varying stimuli show a variety of distinct functions and phenotypes. Classically, it had been thought that an attribution of phenotypic variation in primary DNA structure was explained by a sequence allele or mutation. However, while this theory could explain some aspects of variation, it is difficult to explain how diverse cellular responses are organized under various environmental stimuli.

Epigenetic changes are reversible modifications on DNA or histones and influence the gene expression without altering the sequence of DNA information [91,92]. Epigenetic changes are involved in numerous cellular processes, such as differentiation, immune responses, and tumor development. Based on the knowledge gained from the findings of epigenetic modification, DNA methylation and histone modifications are stable, heritable, and are also reversible processes that influence gene expression with altering chromatin structure. Among epigenetic modifications, DNA methylation occurs at promoter regions, contributing to the repression of gene expression. DNA methylation is known to regulate CADM1 in various tumors, such as ATLL [93], cervical cancer [94,95,96,97], epithelial ovarian cancer [98], oral squamous cell carcinoma [99], and breast cancer [100], and this epigenetic modification-targeted treatment exhibits therapeutic potential against malignancy [93].

In cutaneous solid tumors, the CADM1 promoter is highly methylated in cutaneous melanoma and is also associated with the advance of the tumor stage and disease-related survival methylation, suggesting that CADM1 is an indicator for poor prognoses of melanoma [34]. In addition, long non-coding RNA lymph node metastasis associated transcript 1 (LNMAT1) epigenetically impairs the expression of CADM1 in melanoma by EZH2 recruitment, which is the key enzyme of trimethylation of histone H3 at lysine 27 (H3K27me3) and promotes hypermethylation of the CADM1 promoter, resulting in the transcriptional inhibition of CADM1 [31].

In squamous cell carcinoma, HPV16/18-positive patients with cervical squamous cell carcinoma showed significant differences in the hypermethylation of CADM1 [96]. CADM1 promoter methylation is associated with unfavorable survival rates in patients with oral squamous cell carcinoma [99]. This hypermethylation is expected to be observed in cutaneous squamous cell carcinoma.

### 4.4. The Future Direction of Clinical Application of CADM1-Targeted Therapy

We have summarized possible therapeutic potentials for CADM1-targeted treatment under investigation in current research (Table 2). Several pieces of research conducted a possible therapeutic approach which targeted CADM1. HTLV-1-infected regions gradually advance DNA hypermethylation, indicating the progression of ATLL and contributing to the ATLL leukemogenesis. Inhibition of DNA hypermethylation by a chemical agent, OD-2100, exerts anti-ATLL activity and suppresses tumor growth [93]. Therefore, based on the epigenetic modification of CADM1 by DNA hypermethylation, CADM1-targeted therapy mediated by epigenetic modification will also become a novel therapeutic approach in the future.

Anti-CADM1 antibodies exhibit antibody-dependent cell-mediated cytotoxic activity, and inhibit the interaction between endothelial cells and CADM1-positive ATLL cells [101]. In mice experiments of the transplantation of lymphoma cells expressing CADM1, anti-CADM1 antibodies suppress the organ invasion of lymphoma cells, resulting in improved survival rates.

**Table 2:** Summary of the possible therapeutic potential for CADM1 targeted treatment in cutaneous malignancies.

Cutaneous Malignancies	Possible Therapeutic Options
Solid tumor	
Melanoma	MicroRNA-214 inhibitor [102]
Squamous cell carcinoma	miR-424-5p inhibitor [103]
Lymphoma	
Adult T cell leukemia/lymphoma	OD-2100 [93], anti-CADM1 antibody [101]

As a relationship with cutaneous malignancies, melanoma and squamous cell carcinoma are also candidate diseases for CADM1-targeted therapy. MicroRNA-214 enhances the process of epithelial-mesenchymal transition EMT in melanoma by the downmodulation of CADM1, which is recovered by miR-214 inhibitor, leading to the suppressive effect against EMT processes [102]. In squamous cell carcinoma, CADM1 is a direct target of miR-424-5p, and the overexpression of miR-424-5p promoted tumor development [103]. Therefore, miR-424-5p-targeted indirect suppressive reactions to CADM1 in squamous cell carcinoma might also become a therapeutic tool for the suppression of CADM1 in squamous cell carcinoma.

The expression of CADM1 is low in liver cancer cells and tumor tissues [104]. The lower expression of CADM1 is related with the development of hepatocellular carcinoma. MiR-194 inhibits CADM1 protein level expression in hepatocellular carcinoma (HCC) by inhibiting mRNA translation of CADM1, and promoting proliferation, invasion, migration, and cell cycle progression of HCC cells by inhibition of CADM1 [104]. Therefore, miR-194 might be useful to aid the suppression of CADM1 in the tumor cells. 

## 5. Conclusions

We summarized the role of CADM1 on various cutaneous malignancies. The role of CADM1 is different in bone marrow-derived malignancies and solid tumors, and cutaneous malignancies exhibited different clinical behavior depending on CADM1 expression. These characteristics of CADM1 in cutaneous malignancies basically show the same tendency as the malignancies in other organs. CADM1 acts as adhesion molecules in other cells. Especially, CADM1 contributes to bone marrow-derived cells to the communication with other type cells and promotes cell migration as a scaffolding molecule, which also contributes to cutaneous lymphoma to develop the tumor environment. On the contrary to cutaneous lymphomas, CADM1 contributes to the favorable clinical behavior in solid tumors, except for in Merkel cell carcinoma. Although the reason remains unclear, the characteristics of tumor origins might be associated with the role of CADM1 in tumor development. Skin consists of various components such as eccrine glands, hair follicles, and sensory nerves and receptors, and is therefore problematic. Thus, we cannot predict the CADM1 role in the prognosis of cutaneous tumors simply depending on whether they are solid or bone marrow-derived. Furthermore, the role of CADM1 on other cutaneous malignancies, such as basal cell carcinoma, solar keratosis, and soft tissue malignant tumors, especially angiosarcoma, has not yet been elucidated. Based on the role of CADM1 in the etiology of tumor development, the theory of CADM1 contribution will need to be applied to skin tumors according to other organ malignancies, however, the characteristics of skin as a multicomponent peripheral organ should be kept in mind to conclude prognoses in further investigations. In addition, the regulatory mechanisms of CADM1 in cutaneous malignancies and the usefulness of CADM1 as a biomarker will have to be clarified for therapeutic application in the future. Currently, there are several methods of indirect CADM1-targeted therapy using miRNA, and various aspects of approach will be available in cutaneous malignancies in the future. The incidence of cutaneous malignancy is gradually increasing due to the risk of exposure to ultraviolet light and other environmental stimuli, therefore cutaneous malignancies are highlighted for clinicians. As one of the therapeutic potential targets, CADM1 is expected to become a major investigative field for future advancement of oncological research in the skin and overcome the current limitation of the treatment.

## Figures and Tables

**Table 1:** Summary of the role of CADM1 in cutaneous malignancies.

Cutaneous Malignancies	The Role of CADM1 in the Risk of Each Malignancies
Solid tumor	
Melanoma	Favorable [31,32,33,34].
Squamous cell carcinoma	Favorable [35,36]
Merkel cell carcinoma	Unfavorable [37].
Lymphoma	
Adult T cell leukemia/lymphoma	Unfavorable [10,38].
Mycosis fungoides	Unfavorable [39,40]
Sezary syndrome	Unfavorable [41]

## References

[1] Steinberg M.S. (1996). Adhesion in development: An historical overview. Dev. Biol..

[2] Kuramochi M., Fukuhara H., Nobukuni T., Kanbe T., Maruyama T., Ghosh H.P., Pletcher M., Isomura M., Onizuka M., Kitamura T. (2001). TSLC1 is a tumor-suppressor gene in human non-small-cell lung cancer. Nat. Genet..

[3] Ito A., Okada M., Uchino K., Wakayama T., Koma Y., Iseki S., Tsubota N., Okita Y., Kitamura Y. (2003). Expression of the TSLC1 adhesion molecule in pulmonary epithelium and its down-regulation in pulmonary adenocarcinoma other than bronchioloalveolar carcinoma. Lab. Investig..

[4] Uchino K., Ito A., Wakayama T., Koma Y., Okada T., Ohbayashi C., Iseki S., Kitamura Y., Tsubota N., Okita Y. (2003). Clinical implication and prognostic significance of the tumor suppressor TSLC1 gene detected in adenocarcinoma of the lung. Cancer.

[5] Sasaki H., Nishikata I., Shiraga T., Akamatsu E., Fukami T., Hidaka T., Kubuki Y., Okayama A., Hamada K., Okabe H. (2005). Overexpression of a cell adhesion molecule, TSLC1, as a possible molecular marker for acute-type adult T-cell leukemia. Blood.

[6] Moiseeva E.P., Straatman K.R., Leyland M.L., Bradding P. (2014). CADM1 controls actin cytoskeleton assembly and regulates extracellular matrix adhesion in human mast cells. PLoS ONE.

[7] Biederer T., Sara Y., Mozhayeva M., Atasoy D., Liu X., Kavalali E.T., Südhof T.C. (2002). SynCAM, a synaptic adhesion molecule that drives synapse assembly. Science.

[8] Hasstedt S.J., Bezemer I.D., Callas P.W., Vossen C.Y., Trotman W., Hebbel R.P., Demers C., Rosendaal F.R., Bovill E.G. (2009). Cell adhesion molecule 1: A novel risk factor for venous thrombosis. Blood.

[9] Schwartz R.A., McDonough P.H., Lee B.W. (2013). Toxic epidermal necrolysis: Part II. Prognosis, sequelae, diagnosis, differential diagnosis, prevention, and treatment. J. Am. Acad. Dermatol..

[10] Masuda M., Maruyama T., Ohta T., Ito A., Hayashi T., Tsukasaki K., Kamihira S., Yamaoka S., Hoshino H., Yoshida T. (2010). CADM1 interacts with Tiam1 and promotes invasive phenotype of human T-cell leukemia virus type I-transformed cells and adult T-cell leukemia cells. J. Biol. Chem..

[11] Yageta M., Kuramochi M., Masuda M., Fukami T., Fukuhara H., Maruyama T., Shibuya M., Murakami Y. (2002). Direct association of TSLC1 and DAL-1, two distinct tumor suppressor proteins in lung cancer. Cancer Res..

[12] Shingai T., Ikeda W., Kakunaga S., Morimoto K., Takekuni K., Itoh S., Satoh K., Takeuchi M., Imai T., Monden M. (2003). Implications of nectin-like molecule-2/IGSF4/RA175/SgIGSF/TSLC1/SynCAM1 in cell-cell adhesion and transmembrane protein localization in epithelial cells. J. Biol. Chem..

[13] Sawada Y., Honda T., Hanakawa S., Nakamizo S., Murata T., Ueharaguchi-Tanada Y., Ono S., Amano W., Nakajima S., Egawa G. (2015). Resolvin E1 inhibits dendritic cell migration in the skin and attenuates contact hypersensitivity responses. J. Exp. Med..

[14] Giangreco A., Hoste E., Takai Y., Rosewell I., Watt F.M. (2012). Epidermal Cadm1 expression promotes autoimmune alopecia via enhanced T cell adhesion and cytotoxicity. J. Immunol..

[15] Crivellato E., Ribatti D. (2010). The mast cell: An evolutionary perspective. Biol. Rev. Camb. Philos. Soc..

[16] Otsuka A., Kubo M., Honda T., Egawa G., Nakajima S., Tanizaki H., Kim B., Matsuoka S., Watanabe T., Nakae S. (2011). Requirement of interaction between mast cells and skin dendritic cells to establish contact hypersensitivity. PLoS ONE.

[17] Nakashima C., Ishida Y., Kitoh A., Otsuka A., Kabashima K. (2019). Interaction of peripheral nerves and mast cells, eosinophils, and basophils in the development of pruritus. Exp. Dermatol..

[18] Moiseeva E.P., Leyland M.L., Bradding P. (2013). CADM1 is expressed as multiple alternatively spliced functional and dysfunctional isoforms in human mast cells. Mol. Immunol..

[19] Magadmi R., Meszaros J., Damanhouri Z.A., Seward E.P. (2019). Secretion of Mast Cell Inflammatory Mediators Is Enhanced by CADM1-Dependent Adhesion to Sensory Neurons. Front. Cell. Neurosci..

[20] Ito A., Jippo T., Wakayama T., Morii E., Koma Y., Onda H., Nojima H., Iseki S., Kitamura Y. (2003). SgIGSF: A new mast-cell adhesion molecule used for attachment to fibroblasts and transcriptionally regulated by MITF. Blood.

[21] Sawada Y., Honda T., Nakamizo S., Otsuka A., Ogawa N., Kobayashi Y., Nakamura M., Kabashima K. (2018). Resolvin E1 attenuates murine psoriatic dermatitis. Sci. Rep..

[22] Saito-Sasaki N., Sawada Y., Mashima E., Yamaguchi T., Ohmori S., Yoshioka H., Haruyama S., Okada E., Nakamura M. (2018). Maresin-1 suppresses imiquimod-induced skin inflammation by regulating IL-23 receptor expression. Sci. Rep..

[23] Summerfield A., Meurens F., Ricklin M.E. (2015). The immunology of the porcine skin and its value as a model for human skin. Mol. Immunol..

[24] Jongbloed S.L., Kassianos A.J., McDonald K.J., Clark G.J., Ju X., Angel C.E., Chen C.J., Dunbar P.R., Wadley R.B., Jeet V. (2010). Human CD141+ (BDCA-3) + dendritic cells (DCs) represent a unique myeloid DC subset that cross-presents necrotic cell antigens. J. Exp. Med..

[25] Kaur G., Trowsdale J., Fugger L. (2013). Natural killer cells and their receptors in multiple sclerosis. Brain.

[26] Long E.O., Kim H.S., Liu D., Peterson M.E., Rajagopalan S. (2013). Controlling natural killer cell responses: Integration of signals for activation and inhibition. Annu. Rev. Immunol..

[27] Kabashima K., Honda T., Ginhoux F., Egawa G. (2019). The immunological anatomy of the skin. Nat. Rev. Immunol..

[28] Boles K.S., Barchet W., Diacovo T., Cella M., Colonna M. (2005). The tumor suppressor TSLC1/NECL-2 triggers NK-cell and CD8+ T-cell responses through the cell-surface receptor CRTAM. Blood.

[29] Takahashi S., Ishida A., Kubo A., Kawasaki H., Ochiai S., Nakayama M., Koseki H., Amagai M., Okada T. (2019). Homeostatic pruning and activity of epidermal nerves are dysregulated in barrier-impaired skin during chronic itch development. Sci. Rep..

[30] Perner C., Flayer C.H., Zhu X., Aderhold P.A., Dewan Z.N.A., Voisin T., Camire R.B., Chow O.A., Chiu I.M., Sokol C.L. (2020). Substance P Release by Sensory Neurons Triggers Dendritic Cell Migration and Initiates the Type-2 Immune Response to Allergens. Immunity.

[31] Mou K., Zhang X., Mu X., Ge R., Han D., Zhou Y., Wang L. (2019). LNMAT1 Promotes Invasion-Metastasis Cascade in Malignant Melanoma by Epigenetically Suppressing CADM1 Expression. Front. Oncol..

[32] You Y., Zhang J., Li Y., Li Y., Shi G., Ma L., Wei H. (2014). CADM1/TSLC1 inhibits melanoma cell line A375 invasion through the suppression of matrix metalloproteinases. Mol. Med. Rep..

[33] Hartsough E.J., Weiss M.B., Heilman S.A., Purwin T.J., Kugel C.H., Rosenbaum S.R., Erkes D.A., Tiago M., HooKim K., Chervoneva I. (2019). CADM1 is a TWIST1-regulated suppressor of invasion and survival. Cell. Death Dis..

[34] You Y., Ma L., You M., Li X., Wang S., Li H., Wu D., Yang H., Li Z.Y. (2010). TSLC1 gene silencing in cutaneous melanoma. Melanoma Res..

[35] Liu D., Feng X., Wu X., Li Z., Wang W., Tao Y., Xia Y. (2013). Tumor suppressor in lung cancer 1 (TSLC1), a novel tumor suppressor gene, is implicated in the regulation of proliferation, invasion, cell cycle, apoptosis, and tumorigenicity in cutaneous squamous cell carcinoma. Tumour Biol..

[36] Chahal H.S., Lin Y., Ransohoff K.J., Hinds D.A., Wu W., Dai H.J., Qureshi A.A., Li W.Q., Kraft P., Tang J.Y. (2016). Genome-wide association study identifies novel susceptibility loci for cutaneous squamous cell carcinoma. Nat. Commun..

[37] Iwasaki T., Matsushita M., Nonaka D., Nagata K., Kato M., Kuwamoto S., Murakami I., Hayashi K. (2016). Lower expression of CADM1 and higher expression of MAL in Merkel cell carcinomas are associated with Merkel cell polyomavirus infection and better prognosis. Hum. Pathol..

[38] Dewan M.Z., Takamatsu N., Hidaka T., Hatakeyama K., Nakahata S., Fujisawa J., Katano H., Yamamoto N., Morishita K. (2008). Critical role for TSLC1 expression in the growth and organ infiltration of adult T-cell leukemia cells in vivo. J. Virol..

[39] Mashima E., Sawada Y., Yamaguchi T., Yoshioka H., Ohmori S., Haruyama S., Yoshioka M., Okada E., Nakamura M. (2018). A high expression of cell adhesion molecule 1 (CADM1) is an unfavorable prognostic factor in mycosis fungoides. Clin. Immunol..

[40] Yuki A., Ansai O., Abe R. (2020). CADM1 expression of mast cells in mycosis fungoides. J. Am. Acad. Dermatol..

[41] Yamaguchi M., Morizane S., Hamada T., Miyake T., Sugaya M., Iwata H., Fujii K., Haramoto-Shiratsuki R., Nakagawa Y., Miura M. (2019). The expression of cell adhesion molecule 1 and its splicing variants in Sézary cells and cell lines from cutaneous T-cell lymphoma. J. Dermatol..

[42] Lomas A., Leonardi-Bee J., Bath-Hextall F. (2012). A systematic review of worldwide incidence of nonmelanoma skin cancer. Br. J. Dermatol..

[43] Corchado-Cobos R., García-Sancha N., González-Sarmiento R., Pérez-Losada J., Cañueto J. (2020). Cutaneous Squamous Cell Carcinoma: From Biology to Therapy. Int. J. Mol. Sci..

[44] Burton K.A., Ashack K.A., Khachemoune A. (2016). Cutaneous Squamous Cell Carcinoma: A Review of High-Risk and Metastatic Disease. Am. J. Clin. Dermatol..

[45] Claveau J., Archambault J., Ernst D.S., Giacomantonio C., Limacher J.J., Murray C., Parent F., Zloty D. (2020). Multidisciplinary management of locally advanced and metastatic cutaneous squamous cell carcinoma. Curr. Oncol..

[46] Wu H., Lotan R., Menter D., Lippman S.M., Xu X.C. (2000). Expression of E-cadherin is associated with squamous differentiation in squamous cell carcinomas. Anticancer. Res..

[47] Jenkins R.W., Fisher D.E. (2020). Treatment of Advanced Melanoma in 2020 and Beyond. J. Investig. Dermatol..

[48] Simiczyjew A., Dratkiewicz E., Mazurkiewicz J., Ziętek M., Matkowski R., Nowak D. (2020). The Influence of Tumor Microenvironment on Immune Escape of Melanoma. Int. J. Mol. Sci..

[49] You Y., Wang S.H., Zhang J.F., Zheng S.Y. (2012). TSLC1 expression discriminates cutaneous melanomas from dysplastic nevi. Melanoma Res..

[50] Munhoz de Paula Alves Coelho K., Stall J., Fronza Júnior H., Blasius R., de França P.H.C. (2017). Evaluation of expression of genes CADM1, TWIST1 and CDH1 by immunohistochemestry in melanocytic lesions. Pathol. Res. Pract..

[51] Willemze R., Jaffe E.S., Burg G., Cerroni L., Berti E., Swerdlow S.H., Ralfkiaer E., Chimenti S., Diaz-Perez J.L., Duncan L.M. (2005). WHO-EORTC classification for cutaneous lymphomas. Blood.

[52] Cook L.B., Fuji S., Hermine O., Bazarbachi A., Ramos J.C., Ratner L., Horwitz S., Fields P., Tanase A., Bumbea H. (2019). Revised Adult T-Cell Leukemia-Lymphoma International Consensus Meeting Report. J. Clin. Oncol..

[53] Sawada Y., Hino R., Hama K., Ohmori S., Fueki H., Yamada S., Fukamachi S., Tajiri M., Kubo R., Yoshioka M. (2011). Type of skin eruption is an independent prognostic indicator for adult T-cell leukemia/lymphoma. Blood.

[54] Sawada Y., Shimauchi T., Yamaguchi T., Okura R., Hama-Yamamoto K., Fueki-Yoshioka H., Ohmori S., Yamada S., Yoshizawa M., Hiromasa K. (2013). Combination of skin-directed therapy and oral etoposide for smoldering adult T-cell leukemia/lymphoma with skin involvement. Leuk. Lymphoma.

[55] Tokura Y., Sawada Y., Shimauchi T. (2014). Skin manifestations of adult T-cell leukemia/lymphoma: Clinical, cytological and immunological features. J. Dermatol..

[56] Yamada Y., Tomonaga M., Fukuda H., Hanada S., Utsunomiya A., Tara M., Sano M., Ikeda S., Takatsuki K., Kozuru M. (2001). A new G-CSF-supported combination chemotherapy, LSG15, for adult T-cell leukaemia-lymphoma: Japan Clinical Oncology Group Study 9303. Br. J. Haematol..

[57] Tsukasaki K., Utsunomiya A., Fukuda H., Shibata T., Fukushima T., Takatsuka Y., Ikeda S., Masuda M., Nagoshi H., Ueda R. (2007). VCAP-AMP-VECP compared with biweekly CHOP for adult T-cell leukemia-lymphoma: Japan Clinical Oncology Group Study JCOG9801. J. Clin. Oncol..

[58] Stanchina M., Soong D., Zheng-Lin B., Watts J.M., Taylor J. (2020). Advances in Acute Myeloid Leukemia: Recently Approved Therapies and Drugs in Development. Cancers.

[59] Bladé J., Dimopoulos M., Rosiñol L., Rajkumar S.V., Kyle R.A. (2010). Smoldering (asymptomatic) multiple myeloma: Current diagnostic criteria, new predictors of outcome, and follow-up recommendations. J. Clin. Oncol..

[60] Nakahata S., Saito Y., Marutsuka K., Hidaka T., Maeda K., Hatakeyama K., Shiraga T., Goto A., Takamatsu N., Asada Y. (2012). Clinical significance of CADM1/TSLC1/IgSF4 expression in adult T-cell leukemia/lymphoma. Leukemia.

[61] Vermeer M.H., Nicolay J.P., Scarisbrick J.J., Zinzani P.L. (2020). The importance of assessing blood tumour burden in cutaneous T-cell lymphoma. Br. J. Dermatol..

[62] Quaglino P., Fava P., Pileri A., Grandi V., Sanlorenzo M., Panasiti V., Guglielmo A., Alberti-Violetti S., Novelli M., Astrua C. (2020). Phenotypical Markers, Molecular Mutations, and Immune Microenvironment as Targets for New Treatments in Patients with Mycosis Fungoides and/or Sézary Syndrome. J. Investig. Dermatol..

[63] Wieselthier J.S., Koh H.K. (1990). Sézary syndrome: Diagnosis, prognosis, and critical review of treatment options. J. Am. Acad. Dermatol..

[64] Goessling W., McKee P.H., Mayer R.J. (2002). Merkel cell carcinoma. J. Clin. Oncol..

[65] Heath M., Jaimes N., Lemos B., Mostaghimi A., Wang L.C., Peñas P.F., Nghiem P. (2008). Clinical characteristics of Merkel cell carcinoma at diagnosis in 195 patients: The AEIOU features. J. Am. Acad. Dermatol..

[66] Babadzhanov M., Doudican N., Wilken R., Stevenson M., Pavlick A., Carucci J. (2020). Current concepts and approaches to merkel cell carcinoma. Arch. Dermatol. Res..

[67] Kaufman H.L., Russell J., Hamid O., Bhatia S., Terheyden P., D’Angelo S.P., Shih K.C., Lebbé C., Linette G.P., Milella M. (2016). Avelumab in patients with chemotherapy-refractory metastatic Merkel cell carcinoma: A multicentre, single-group, open-label, phase 2 trial. Lancet Oncol..

[68] Qian J.B., Liu H.B., Zhu Y., Lu F., Yang Q.C., Shen Y. (2017). CADM1 mRNA expression and clinicopathological significance in esophageal squamous cell carcinoma tissue. Genet. Mol. Res..

[69] Tsuboi Y., Oyama M., Kozuka-Hata H., Ito A., Matsubara D., Murakami Y. (2020). CADM1 suppresses c-Src activation by binding with Cbp on membrane lipid rafts and intervenes colon carcinogenesis. Biochem. Biophys. Res. Commun..

[70] Saito M., Goto A., Abe N., Saito K., Maeda D., Ohtake T., Murakami Y., Takenoshita S. (2018). Decreased expression of CADM1 and CADM4 are associated with advanced stage breast cancer. Oncol. Lett..

[71] Chen Y., Liu L., Guo Z., Wang Y., Yang Y., Liu X. (2019). Lost expression of cell adhesion molecule 1 is associated with bladder cancer progression and recurrence and its overexpression inhibited tumor cell malignant behaviors. Oncol. Lett..

[72] Si X., Xu F., Xu F., Wei M., Ge Y., Chenge S. (2020). CADM1 inhibits ovarian cancer cell proliferation and migration by potentially regulating the PI3K/Akt/mTOR pathway. Biomed. Pharmacother..

[73] Kato T., Hagiyama M., Takashima Y., Yoneshige A., Ito A. (2018). Cell adhesion molecule-1 shedding induces apoptosis of renal epithelial cells and exacerbates human nephropathies. Am. J. Physiol. Ren. Physiol..

[74] Hagiyama M., Kimura R., Yoneshige A., Inoue T., Otani T., Ito A. (2020). Cell Adhesion Molecule 1 Contributes to Cell Survival in Crowded Epithelial Monolayers. Int. J. Mol. Sci..

[75] Sun S., Liu W., Li Y. (2020). CADM1 enhances intestinal barrier function in a rat model of mild inflammatory bowel disease by inhibiting the STAT3 signaling pathway. J. Bioenerg. Biomembr..

[76] Kobayashi M., Shu S., Marunaka K., Matsunaga T., Ikari A. (2020). Weak Ultraviolet B Enhances the Mislocalization of Claudin-1 Mediated by Nitric Oxide and Peroxynitrite Production in Human Keratinocyte-Derived HaCaT Cells. Int. J. Mol. Sci..

[77] Furuse M., Hata M., Furuse K., Yoshida Y., Haratake A., Sugitani Y., Noda T., Kubo A., Tsukita S. (2002). Claudin-based tight junctions are crucial for the mammalian epidermal barrier: A lesson from claudin-1-deficient mice. J. Cell. Biol..

[78] Bergmann S., von Buenau B., Vidal Y.S.S., Haftek M., Wladykowski E., Houdek P., Lezius S., Duplan H., Bäsler K., Dähnhardt-Pfeiffer S. (2020). Claudin-1 decrease impacts epidermal barrier function in atopic dermatitis lesions dose-dependently. Sci. Rep..

[79] Rathjen T., Yan X., Kononenko N.L., Ku M.C., Song K., Ferrarese L., Tarallo V., Puchkov D., Kochlamazashvili G., Brachs S. (2017). Regulation of body weight and energy homeostasis by neuronal cell adhesion molecule 1. Nat. Neurosci..

[80] Yan X., Kononenko N.L., Brüel A., Thomsen J.S., Poy M.N. (2018). Neuronal Cell Adhesion Molecule 1 Regulates Leptin Sensitivity and Bone Mass. Calcif. Tissue Int..

[81] Ito T., Nakamura A., Tanaka I., Tsuboi Y., Morikawa T., Nakajima J., Takai D., Fukayama M., Sekido Y., Niki T. (2019). CADM1 associates with Hippo pathway core kinases; membranous co-expression of CADM1 and LATS2 in lung tumors predicts good prognosis. Cancer Sci..

[82] Murakami S., Sakurai-Yageta M., Maruyama T., Murakami Y. (2014). Trans-homophilic interaction of CADM1 activates PI3K by forming a complex with MAGuK-family proteins MPP3 and Dlg. PLoS ONE.

[83] Qian Y., Corum L., Meng Q., Blenis J., Zheng J.Z., Shi X., Flynn D.C., Jiang B.H. (2004). PI3K induced actin filament remodeling through Akt and p70S6K1: Implication of essential role in cell migration. Am. J. Physiol. Cell. Physiol..

[84] Gouëffic Y., Guilluy C., Guérin P., Patra P., Pacaud P., Loirand G. (2006). Hyaluronan induces vascular smooth muscle cell migration through RHAMM-mediated PI3K-dependent Rac activation. Cardiovasc. Res..

[85] Lien E.C., Dibble C.C., Toker A. (2017). PI3K signaling in cancer: Beyond AKT. Curr. Opin. Cell. Biol..

[86] Nakahata S., Syahrul C., Nakatake A., Sakamoto K., Yoshihama M., Nishikata I., Ukai Y., Matsuura T., Kameda T., Shide K. (2020). Clinical significance of soluble CADM1 as a novel marker for adult T-cell leukemia/lymphoma. Haematologica.

[87] Makiyama J., Kobayashi S., Watanabe E., Ishigaki T., Kawamata T., Nakashima M., Yamagishi M., Nakano K., Tojo A., Watanabe T. (2019). CD4(+) CADM1(+) cell percentage predicts disease progression in HTLV-1 carriers and indolent adult T-cell leukemia/lymphoma. Cancer Sci..

[88] Rong G., Zhang M., Xia W., Li D., Miao J., Wang H. (2019). Plasma CADM1 promoter hypermethylation and D-dimer as novel metastasis predictors of cervical cancer. J. Obstet. Gynaecol. Res..

[89] Hagiyama M., Nakatani Y., Takashima Y., Kato T., Inoue T., Kimura R., Otani T., Sato Y., Mori H., Arima S. (2019). Urinary Cell Adhesion Molecule 1 Is a Novel Biomarker That Links Tubulointerstitial Damage to Glomerular Filtration Rates in Chronic Kidney Disease. Front. Cell. Dev. Biol..

[90] Crick F. (1970). Central dogma of molecular biology. Nature.

[91] Sawada Y., Gallo R. (2020). Role of epigenetics in the regulation of immune functions of the skin. J. Investig. Dermatol..

[92] Cavalli G., Heard E. (2019). Advances in epigenetics link genetics to the environment and disease. Nature.

[93] Watanabe T., Yamashita S., Ureshino H., Kamachi K., Kurahashi Y., Fukuda-Kurahashi Y., Yoshida N., Hattori N., Nakamura H., Sato A. (2020). Targeting aberrant DNA hypermethylation as a driver of ATL leukemogenesis by using the new oral demethylating agent OR-2100. Blood.

[94] Yanatatsaneejit P., Chalertpet K., Sukbhattee J., Nuchcharoen I., Phumcharoen P., Mutirangura A. (2020). Promoter methylation of tumor suppressor genes induced by human papillomavirus in cervical cancer. Oncol. Lett..

[95] Holubekova V., Mersakova S., Grendar M., Snahnicanova Z., Kudela E., Kalman M., Lasabova Z., Danko J., Zubor P. (2020). The Role of CADM1 and MAL Promoter Methylation in Inflammation and Cervical Intraepithelial Neoplasia. Genet. Test. Mol. Biomark..

[96] Meršaková S., Holubeková V., Grendár M., Višňovský J., Ňachajová M., Kalman M., Kúdela E., Žúbor P., Bielik T., Lasabová Z. (2018). Methylation of CADM1 and MAL together with HPV status in cytological cervical specimens serves an important role in the progression of cervical intraepithelial neoplasia. Oncol. Lett..

[97] Fiano V., Trevisan M., Fasanelli F., Grasso C., Marabese F., da Graça Bicalho M., de Carvalho N.S., Maestri C.A., Merletti F., Sacerdote C. (2018). Methylation in host and viral genes as marker of aggressiveness in cervical lesions: Analysis in 543 unscreened women. Gynecol. Oncol..

[98] Hassan Z.K., Hafez M.M., Kamel M.M., Zekri A.R. (2017). Human Papillomavirus Genotypes and Methylation of CADM1, PAX1, MAL and ADCYAP1 Genes in Epithelial Ovarian Cancer Patients. Asian Pac. J. Cancer Prev..

[99] Ribeiro I.P., Caramelo F., Esteves L., Oliveira C., Marques F., Barroso L., Melo J.B., Carreira I.M. (2018). Genomic and epigenetic signatures associated with survival rate in oral squamous cell carcinoma patients. J. Cancer.

[100] Vermeulen M.A., van Deurzen C.H.M., Doebar S.C., de Leng W.W.J., Martens J.W.M., van Diest P.J., Moelans C.B. (2019). Promoter hypermethylation in ductal carcinoma in situ of the male breast. Endocr. Relat. Cancer.

[101] Chilmi S., Nakahata S., Fauzi Y.R., Ichikawa T., Tani C., Suwanruengsri M., Yamaguchi R., Matsuura T., Kurosawa G., Morishita K. (2020). Development of anti-human CADM1 monoclonal antibodies as a potential therapy for adult T-cell leukemia/lymphoma. Int. J. Hematol..

[102] Wang S.J., Li W.W., Wen C.J., Diao Y.L., Zhao T.L. (2020). MicroRNA-214 promotes the EMT process in melanoma by downregulating CADM1 expression. Mol. Med. Rep..

[103] Li Y., Liu J., Hu W., Zhang Y., Sang J., Li H., Ma T., Bo Y., Bai T., Guo H. (2019). MiR-424-5p Promotes Proliferation, Migration and Invasion of Laryngeal Squamous Cell Carcinoma. Onco Targets Ther..

[104] Niu X., Nong S., Gong J., Zhang X., Tang H., Zhou T., Li W. (2020). MiR-194 promotes hepatocellular carcinoma through negative regulation of CADM1. Int. J. Clin. Exp. Pathol..

